# Cumulative early life adversity predicts longevity in wild baboons

**DOI:** 10.1038/ncomms11181

**Published:** 2016-04-19

**Authors:** Jenny Tung, Elizabeth A. Archie, Jeanne Altmann, Susan C. Alberts

**Affiliations:** 1 Department of Evolutionary Anthropology, Box 90383, Duke University, Durham, North Carolina 27708, USA; 2 Duke Population Research Institute, Box 90989, Duke University, Durham, North Carolina 27708, USA; 3 Institute of Primate Research, National Museums of Kenya, PO Box 24481, Nairobi 00502, Kenya; 4 Department of Biological Sciences, University of Notre Dame, 100 Galvin Life Science Center, Notre Dame, Indiana 46556, USA; 5 Department of Ecology and Evolutionary Biology, Princeton University, 106A Guyot Hall, Princeton, New Jersey 08544, USA; 6 Department of Veterinary Anatomy and Physiology, PO Box 30197, University of Nairobi, Nairobi 00100, Kenya; 7 Department of Biology, Box 90338, Duke University, Durham, North Carolina 27708, USA

## Abstract

In humans and other animals, harsh circumstances in early life predict morbidity and mortality in adulthood. Multiple adverse conditions are thought to be especially toxic, but this hypothesis has rarely been tested in a prospective, longitudinal framework, especially in long-lived mammals. Here we use prospective data on 196 wild female baboons to show that cumulative early adversity predicts natural adult lifespan. Females who experience ≥3 sources of early adversity die a median of 10 years earlier than females who experience ≤1 adverse circumstances (median lifespan is 18.5 years). Females who experience the most adversity are also socially isolated in adulthood, suggesting that social processes partially explain the link between early adversity and adult survival. Our results provide powerful evidence for the developmental origins of health and disease and indicate that close ties between early adversity and survival arise even in the absence of health habit and health care-related explanations.

## Abstract (web summary)

Adverse early life experiences can have negative consequences for health and survival in later life. Here, Tung *et al*. show that in female baboons, the cumulative number of adverse experiences can be a strong predictor of lifespan.

## Graphical abstract

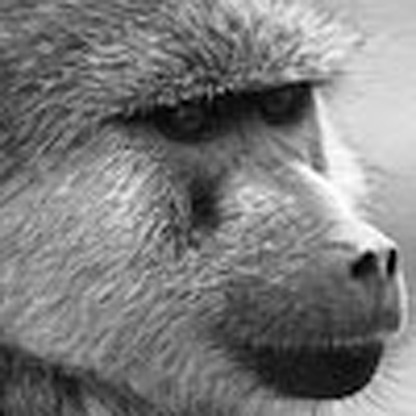

Adverse conditions in early life are thought to have far-reaching implications for adult health and survival. In humans, the strongest evidence for these effects comes from studies that link early adversity to the risk of cardiovascular disease, schizophrenia and type II diabetes in adulthood^1^,^2^,^3^,^4^,^5^. Such associations may contribute to the fundamental health disparities that arise from low socioeconomic status, child abuse and major disasters such as war or famine^1^,^6^,^7^,^8^,^9^. However, the roots of early life effects likely reach much further back in our evolutionary history. In non-human animals, early adversity has also been shown to predict components of fitness^10^,^11^,^12^,^13^, and in long-lived vertebrates many studies have found a relationship between measures of early adversity and adult fertility^10^,^14^,^15^,^16^,^17^.

The parallels between early life effects in humans and natural animal populations suggest that animal models can be used to test the generality of conceptual frameworks proposed for humans. In humans, the cumulative adversity model argues that health and survival inequalities begin with vulnerabilities *in utero* and widen as a result of differential exposure to environmental hazards during infancy and childhood^18^,^19^,^20^. The accumulation of adverse conditions in the first few years of life thus sets individuals onto distinct biological and social tracks that have enduring consequences for later life morbidity and mortality^5^,^20^,^21^. While this framework is compelling, studies that test these ideas using prospective, longitudinal data are rare. Moreover, such data have never been used to test the cumulative effects of multiple, diverse and directly observed environmental insults on the length of natural adult lifespans in wild animals (prospective studies in humans are underway, for example, refs 1, 22, 23).

Our objective in this study was to test the correlation between cumulative early adversity and adult longevity in a natural population of baboons, where long-term observations of individually recognized animals provide data on the occurrence of multiple adverse circumstances starting from birth. Our focus on lifespan is relevant to understanding both healthy aging in humans and sources of variance in a major component of fitness. To investigate whether social processes contribute to the correlation between early adversity and longevity, we also investigated whether early adversity predicts social integration in adulthood, a known predictor of female longevity in humans, baboons and several other social mammals^24^,^25^,^26^,^27^,^28^. Our findings support the idea that adverse early events act in aggregate to profoundly influence adult survival, even in the absence of variation in the health risk behaviours and health care access thought to mediate these effects in humans. Specifically, females who experience more cumulative early adversity have significantly shorter adult lifespans—on the order of years—which translates into fewer surviving offspring and lower lifetime reproductive success. Interestingly, they are also more socially isolated from other females as adults, but not from adult males. Together, these results encourage renewed attention to the evolutionary origins and mechanistic basis of the developmental origins of health and disease.

## Results

### Individual sources of early adversity and adult lifespan

To test the relationship between early life adversity and adult mortality, we drew on data from the Amboseli baboon population in southern Kenya. This population has been monitored continuously since 1971, when the Amboseli Baboon Research Project began collecting data on the demography and behaviour of individually recognized baboons on a near-daily basis. Consequently, all adult females in our study (*n*=196; *n*=73 with complete life histories) were prospectively observed since birth, with birth dates accurate to within a few days' error. Following Lindstrom^10^, we defined ‘early life' as the period from conception to reproductive maturity and investigated major sources of environmental adversity that occurred during this time period. Because we were particularly interested in the long-term effects of early adversity, we examined mortality risk during adulthood (that is, after the end of the ‘early life' period), starting at 4 years of age, the earliest age of adulthood (females in Amboseli typically attain menarche between 4 and 5 years^29^).

For each female, we considered exposure to six potential sources of early life adversity: (1) drought in the first year of life, which compromises female fertility^30^,^31^; (2) experienced density, measured as group size at birth, because group size captures competition among group members and predicts female fertility^30^,^32^,^33^,^34^; (3) maternal dominance rank, which is linked to growth rates and maturation timing in offspring^32^,^33^,^35^; (4) maternal affiliative social connectedness, which predicts both maternal and offspring survival^24^,^25^,^36^; (5) maternal loss before age 4 years, because mothers represent a primary social resource for their daughters, even after nutritional independence^37^,^38^; and (6) the presence of a competing younger sibling born <1.5 years after the female's own birth, which may divert maternal investment in the older focal offspring^39^. While some of these sources of adversity may act through common mechanisms (for example, food limitation), they exhibit very different structure in our population. For example, experienced density varies across social groups, rainfall varies temporally, and maternal social connectedness varies among individuals within the same group (note that the absence of reproductive seasonality in baboons means that strong ‘cohort' effects tend to be uncommon; because of high levels of social and environmental variability, even within years, individuals born close in time may have very different early life experiences). Thus, females in our population varied in their exposure to these risk factors, and, unlike studies of cumulative risk in humans^1^,^6^,^40^, most of the risk variables were not correlated with each other (Supplementary Table 1). Our analyses therefore examine largely distinct effects of early adversity on adult survival (see also Supplementary Note 1).

To test the relationship between early adversity and adult longevity, we first constructed a Cox proportional hazards model that modelled each source of adversity as a separate fixed effect (hereafter called the ‘individual effects' model). This model explained 11% of the total variance in female longevity (Wald test, *P*=3.802 × 10^−4^, *n*=196 females; Akaike Information Criterion, AIC=600.74). However, of the six sources of adversity, only maternal loss and the presence of a competing sibling were significant (Supplementary Table 2). Adult females who lost their mothers were three times as likely to die at every age (*P*=3.03 × 10^−5^, *n*=196 females; hazard ratio=3.01; 95% confidence interval (CI)=(1.80, 5.04); Supplementary Table 2), and females with close-in-age younger siblings were almost twice as likely to die at every age as females without competing siblings (*P*=0.030, *n*=196 females; hazard ratio=1.97; 95% CI=(1.07, 3.61); Supplementary Table 2). There were no detectable relationships between adult survival and the other four sources of adversity in this analysis (Supplementary Table 2), although parameter estimates for all four variables were in the predicted directions (shorter lifespan with lower maternal rank, lower maternal social connectedness, first year drought and larger group size).

### Cumulative early adversity and adult lifespan

The results of the individual effects model suggest that the correlation between early adversity and adult longevity is relatively simple for female baboons and is primarily driven by maternal investment. This inference contrasts with the predictions of the cumulative adversity hypothesis, which proposes that adverse environmental conditions act in aggregate to influence adult health and survival^1^,^6^,^41^. To test for cumulative effects, we constructed a simple cumulative adversity index by summing the number of adverse conditions each female experienced early in her life. Our strategy was based on analyses of early adversity and allostatic load (that is, cumulative wear and tear^42^,^43^) in human populations, in which the sum of insults experienced has proven more predictive than modelling each variable independently^6^,^40^,^41^,^44^. For continuous variables, adverse conditions were binarized and defined as those that fell in the lowest-ranking quartile of ordinal ranks, lowest quartile of social connectedness, or largest quartile of group sizes, respectively, relative to the population distribution (following, for example, refs 44, 45). A similar proportion of females experienced early maternal loss (25%), had a competing younger sibling (18.4%), or was born during a drought (18.4%). Thus, a female who experienced an early life insult was approximately equally likely to have been affected by any of the six sources of adversity we considered, ensuring that our analyses were not driven by a subset of common adverse conditions.

**Figure 1:**
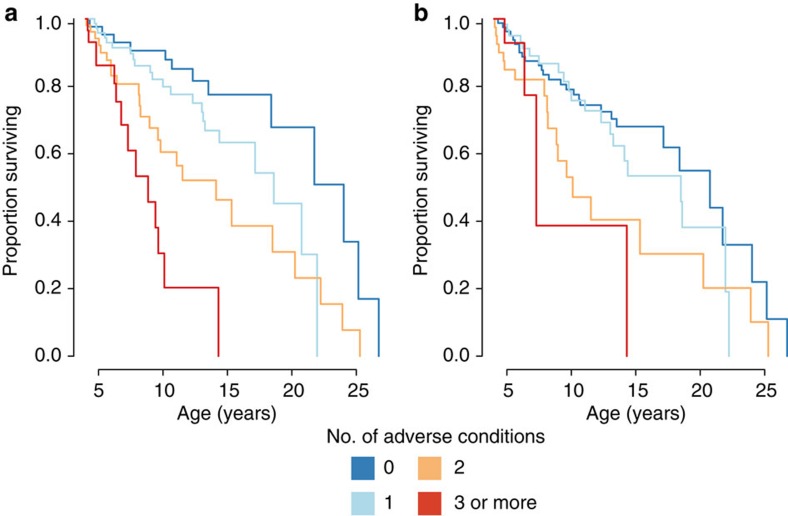
Effect of cumulative early adversity on lifespan in female baboons. (**a**) Survival curves using the full six factor cumulative adversity index (Wald test *P*=7.75 × 10^−7^, *N*=196); (**b**) Survival curves using a reduced four factor adversity index, without the effects of early maternal loss and competing younger sibling (Wald test *P*=0.004, *N*=196). Colours indicate the number of adverse conditions occurring in early life.

**Figure 2:**
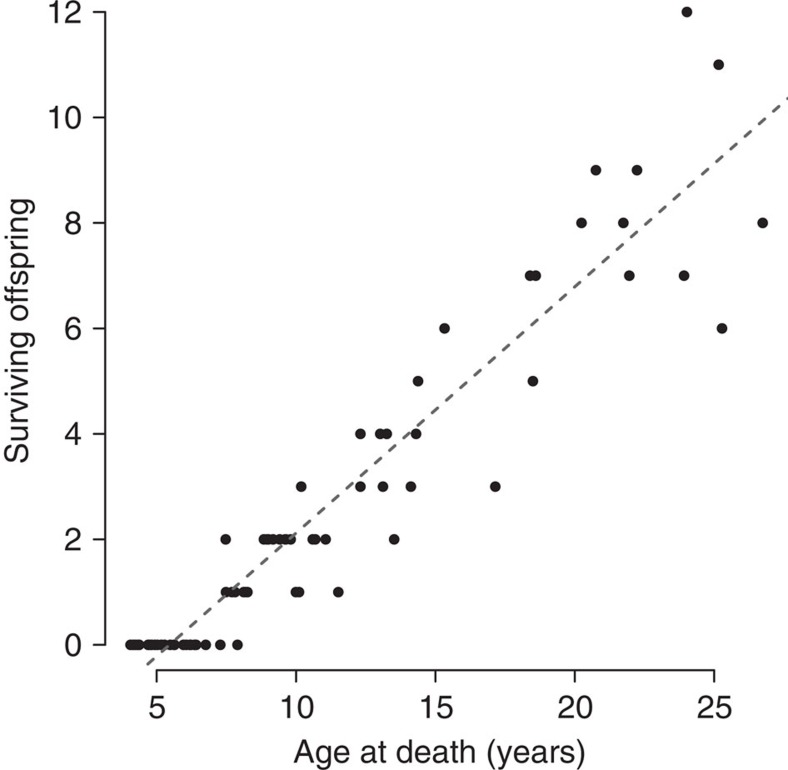
Relationship between lifespan and reproductive success. Female baboons in Amboseli produce a single surviving offspring once every 2.13 years on average (based on the slope of the regression line), and do not exhibit extended post-reproductive lifespans. Surviving offspring are defined here as offspring who lived to at least age 1 (note that inter-birth intervals thus differ from estimates based on all offspring produced, which we used to define competing younger siblings). Among females in our data set who reached adulthood, lifespan explains 89.5% of the variance in the total number of surviving offspring (Pearson's *r*=0.946, *P*<2.2 × 10^−16^, *N*=72); thus, survival is a strong predictor of lifetime reproductive success.

Early life experiences for female baboons in Amboseli were highly variable. Forty-three females in our data set experienced none of the six adverse conditions; 72 experienced only one; 52 experienced two; and 29 experienced three or more (23 experienced 3, and 6 experienced 4 risk factors; none experienced 5 or 6). The cumulative adversity model strongly predicted lifespan (Wald test *P*=7.75 × 10^−7^, *n*=196 females; Fig. 1a), with better support than the individual effects model (*r*^2^=0.12, AIC=589.32 versus *r*^2^=0.11, AIC=600.74). The addition of a single source of adversity nearly doubled the estimated risk of death at every adult age (hazard ratio=1.90; 95% CI=(1.474, 2.453)), translating into a striking difference in median survival. Specifically, among females that experienced ≥3 sources of adversity, more than 50% were predicted to die by age 9 (median survival=8.85 years), while females who experienced 0 or 1 source(s) of adversity had predicted median survival times of 24.02 and 18.60 years, respectively—over 10 years longer (median survival for all females in the data set was 18.5 years, based on the background hazard function; maximum observed lifespan was 26.7 years). Unlike in humans, female baboons do not have a long post-reproductive lifespan^46^, suggesting that differences in longevity have major consequences for lifetime reproductive success. Indeed, when considering only those females in our data set with complete life histories, we identified a striking correlation between age at death and the total number of offspring that survived until at least age 1 (*n*=73, *r*=0.946, *P*<2.2 × 10^−16^; Fig. 2). In other words, females exposed to high adversity early in life paid a cost both in years of their own lives and in lifetime reproductive success.

To ensure that the results of the cumulative adversity model were not solely driven by maternal loss and close-in-age siblings, the two significant effects in the individual effects model, we recalculated the cumulative adversity index excluding these two predictors. This reduced cumulative adversity index also significantly predicted female lifespan, albeit with less explanatory power (Fig. 1b; *r*^2^=0.04; Wald test *P*=0.004, *n*=196 females; hazard ratio=1.47; 95% CI=(1.133, 1.893)). In this case, median predicted ages at death for females who experienced no adversity or one source of adversity were 20.76 and 18.50 years, respectively, compared with 7.28 years for females with a cumulative adversity index ≥3 (Fig. 1b). Importantly, the cumulative adversity approach also outperformed an individual effects model when predictor variables were coded identically in both analyses. Specifically, if we binarized the data for each source of adversity using the same criteria as in the cumulative adversity model, the binarized individual effects model still received less statistical support than the cumulative adversity model (AIC=589.32 versus AIC=594.96; Supplementary Table 3). In addition, prediction error was lowest for the cumulative adversity model compared with the other models we considered (integrated Brier score=0.146 for the cumulative adversity model, 0.158 for the individual effects model and 0.150 for the individual effects model with binary terms; Supplementary Fig. 1). Thus, the cumulative adversity model captures contingent, non-linear effects not captured in the individual effects model analyses.

### Cumulative early adversity predicts adult social integration

In humans, early life adversity is thought to act in part by touching off a cascade of adverse circumstances throughout life, especially social disadvantages, which may further compromise health^5^,^47^. In particular, early adversity may be correlated with poor social environments in adulthood, which are known predictors of adult health and survival^26^. Prior research in Amboseli has found that social connectedness to females and males independently predict adult female longevity^24^. Specifically, adult females who are socially isolated from adult females, adult males or both sexes, lead shorter lives than females who have strong social connectedness to one or both sexes^24^,^25^. We therefore investigated whether a female's social connectedness to adult females or to adult males was predicted by the early life cumulative adversity index (controlling for other known predictors of social connectedness: age, dominance rank and availability of adult female kin^24^).

**Figure 3:**
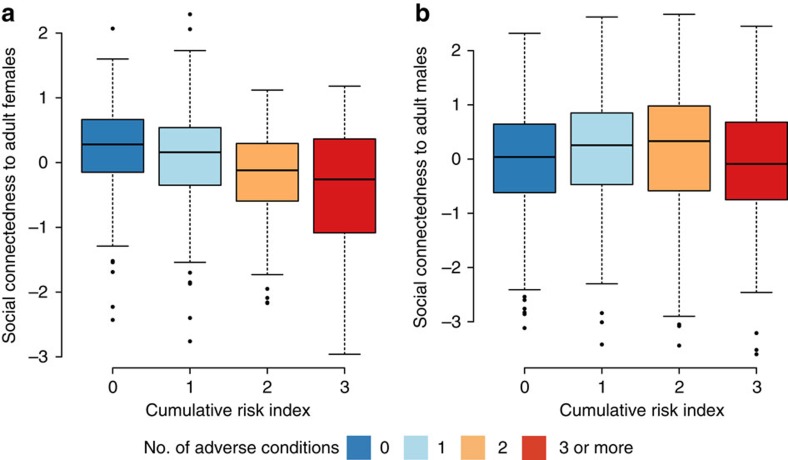
Effect of early adversity on social connectedness during adulthood in female baboons. Adult females that experienced high levels of early adversity were more socially isolated from (**a**) other adult females (LMM: *P*=0.005, *n*=123), but not from (**b**) adult males (LMM: *P*=0.503, *n*=123). The index of social connectedness on the *y* axis reflects the strength of female social connectedness relative to all other females alive in the population in the same year. High values indicate relatively high social connectedness, and low values indicate relative social isolation. Colours indicate the number of adverse conditions occurring in early life. Heavy lines in the middle of each coloured box mark the median, the top and bottom edges mark the 25th and 75th percentiles, and whiskers are the largest or smallest values at 1.5 times the interquartile range.

We found that cumulative early life adversity predicted the strength of a female's social connectedness to other adult females (Fig. 3a; Supplementary Table 4), but not to adult males (Fig. 3b; Supplementary Table 5). Specifically, females who experienced high levels of early life adversity were more socially isolated from other females during adulthood than females who experienced little early adversity (linear mixed model (LMM): *β*=−0.14, *P*=0.005, *n*=123). This effect was not solely driven by females' relationships with their mothers, as removing maternal social connectedness or maternal loss as components of the cumulative adversity index still produced a significant negative relationship between cumulative early adversity and adult female social connectedness (LMM without maternal social connectedness: *β*=−0.13, *P*=0.025; LMM without maternal loss: *β*=−0.16, *P*=0.002, *n*=123).

## Discussion

Our results support the idea that cumulative insults in early life strongly predict survival. While cumulative effects of early adversity have long been hypothesized, our analysis represents the first prospective study to test for a correlation between cumulative adversity (measured as aggregate exposure to multiple early life insults) and natural lifespan in any species, including humans (where studies thus far have been either retrospective or cross-sectional^45^,^46^,^47^). Our results extend previous findings in wild large mammals that show a relationship between survival and physiological proxies of early life insults (for example, reduced body mass^17^) by directly connecting survival with the specific environmental exposures themselves. Together, our findings contribute to three important insights.

First, they indicate the utility of using a cumulative risk approach to investigate aggregate early life effects, especially in small data sets. By doing so, we were able to identify contributions of early life insults that were undetectable when modelling each insult individually. Indeed, although the results of the cumulative risk model support complex interactions between early life conditions, no interactions involving the two best-supported variables in the independent effects model (maternal loss and competing younger sibling) and any other early life insult were statistically detectable in our data set (all *P* values were >0.1). The ability of index approaches to capture such effects without needing to explicitly model all pairwise (or higher order) interactions has been noted in studies of human populations^40^,^41^, but have not been applied to study naturally aging animal populations. Our results indicate the potential benefits of doing so.

Second, our findings connect cumulative early adversity to the length of natural adult lifespans, and hence the survival component of fitness. Further, female baboons that suffered high levels of early adversity not only lived vastly shortened lives, but also experienced an enormous cost to their potential reproductive output (Fig. 2). Specifically, because female baboons, like other nonhuman primates, reproduce throughout the life and rarely experience a post-reproductive life^46^,^48^, the lifespan differences experienced by females with different levels of early adversity translated into substantial differences in lifetime reproductive success. Similar patterns have been found in other long-lived species (for example, refs 49, 50, 51). Our results thus suggest that the scope for adaptive responses to early adversity is limited. Notably, baboons are a socially complex species that inhabits a savanna environment similar to that exploited by early hominins^52^,^53^. Hence, early life effects on health in humans today may be the modern equivalent of early life effects on fitness components that have been important throughout much of human evolutionary history—and not a consequence of the modern human environment, as some hypotheses propose (for example, refs 54, 55, 56).

Third, our results provide some clues about the mechanisms that underlie early life effects on longevity. While some of the effects we observed are likely to be mediated by resource limitation, they also appear to act in part through the relationship between early adversity and adult social relationships. Indeed, adverse social conditions are strongly correlated with morbidity and mortality outcomes in humans and other animals, including in Amboseli, where social isolation in adulthood predicts shorter lifespans for female baboons on the order of 2–3 years^24^. Our results extend these observations to suggest that early adversity predicts adult social isolation. Notably, however, early adversity was only correlated with female social isolation from other adult females, and not from adult males, suggesting that forming strong relationships with adult males might be a route through which females can mitigate the negative effects of early life insults. We do not yet understand why females who experience more adversity in early life find it challenging to establish strong affiliative relationships with other adult females, but not adult males. However, regardless of the causes, our observation lends support to the argument that relationships between social adversity in adulthood, and health and survival outcomes may be partly driven by early life events^57^. Future work will be important in disentangling these effects, and especially for dissecting the relative importance of a path connecting early adversity to longevity via social relationships versus other, independent mechanisms (for example, resource mediation).

## Methods

### Study population and data collection

All demographic, behavioural and ecological data for this study were collected between 1983 and 2013 as part of longitudinal monitoring of wild baboons (*Papio cynocephalus*) in the Amboseli basin of southern Kenya (approximately 1,700 animals since 1983, 250–350 of all ages alive at any given time). Permission to conduct this research was granted by the Kenya Wildlife Service, the Kenyan governmental body that oversees wildlife (most recent permit numbers NACOSTI/P/15/8999/7886 to JT, NACOSTI/P/14/2921/3128 to EAA, NCST/RCD/12B/012/55 to JA, and NACOSTI/P/15/1973/7878 to SCA) and overseen by Institutional Animal Care and Usage Committee protocol approvals at Duke University (A020-15-01), University of Notre Dame (13-11-1352) and Princeton University (1944-13).

Subjects were individually known based on visual recognition. All subjects were habituated to the presence of neutral observers, who followed 1–2 social groups of baboons each day, 6 days a week, alternating mornings and afternoons, year round. Demographic data, including births and disappearances, were collected via systematic group censuses. Because female baboons remain in the groups in which they were born throughout their lives (barring group fissions, which are also captured in our observational data), female disappearances can be reliably attributed to deaths. Hence, all females in the study had birth and death dates accurate to within a few days' error. We did not include male baboons in our study because they routinely transfer between groups, making male disappearances ambiguous (they could be either deaths or transfers).

Of the females monitored during this period, we retained only females who met the following criteria: (1) they survived to age 4 years, which is near the earliest age of adulthood in this population (median age at menarche is 4.5 years; 80% of females reach menarche between 4 and 5 years, and only 7% reach menarche before the age of 4 years^29^); (2) they were members of groups that foraged exclusively on wild foods, and were not members of any of the ‘Lodge' groups monitored in this population, who exploit refuse from human tourists for part of their diet; and (3) data were available for all six sources of early life adversity we considered. Importantly, females who were retained in the analysis were not a biased sample of the study population: in a Cox proportional hazards model, females who satisfied criteria 1 and 2 but had missing data on one or more sources of early adversity (*n*=281) did not differ in survival time from the females included in the final data set (Wald test *P*=0.406; hazard ratio=0.864; 95% CI=(0.611, 1.22)).

The final data set included 196 known-aged female baboons, born into eight different social groups. Seventy-three of these individuals were followed from birth until death; life histories for the remaining 123 were right-censored (of these females, 119 were still alive at the last census date and 4 were censored because of changes in group monitoring during the long-term study). The sources of early life adversity we considered were defined as follows:

- Drought was defined as ≤200 mm of cumulative rainfall in the first year of life. Rainfall is measured daily at the field site using a rain gauge and varies substantially across hydrological years (Supplementary Fig. 2). We chose to model drought as a binary variable based on prior studies of drought effects in Amboseli^30^,^31^, in which using a categorical threshold—when food and water truly seem to become resource limiting—was a better predictor of physiological consequences for female baboons than using a continuous measure of rainfall.
- Experienced density was measured as the number of adults of both sexes in an individual's social group on the day that individual was born. The number of adults (post-menarcheal females and males with enlarged testes) in a group on a given day is based on regular complete censuses on each monitoring day. Group density is highly stable from month to month (for example, using density on the day of birth is highly correlated with mean density in the 3 months surrounding birth: *r* ^2^=0.99).
- Maternal dominance rank was defined as the ordinal dominance rank of the female's mother in the month that the female was born. Dominance ranks were assigned monthly based on the outcomes of dyadic aggressive encounters that are recorded using *ad libitum* observations^58^. Because dominance hierarchies in female baboons are linear and transitive, ordinal ranks can be obtained by minimizing the number of interactions in which higher ranking females lose interactions with lower ranking females.
- Maternal social connectedness was measured as the social connectedness of a female's mother to other adult females. Specifically, maternal social connectedness was calculated as the subject's mother's average value over the first 2 years of the subject's life. Our measures of social connectedness are based on a standardized index that measures the frequency with which the female's mother was a grooming partner with other adult females in her social group, relative to grooming rates for all adult females alive in the population in the same year and adjusted for observer effort^24^.
- Maternal loss was based on the observation of a mother's death before 4 years of age. Young baboons become nutritionally independent from their mothers around 1.5 year of age^59^. However, juveniles remain socially dependent on their mothers, who are their primary defenders and social partners, for much longer. Age 4 corresponds approximately to the earliest age at menarche, when a female may start investing in offspring of her own^29^.
- Presence of a competing younger sibling was assessed using birthdates for the focal female and her next younger sibling, collected in the census data. The birth of a sibling may reduce maternal investment in her daughter, especially if the sibling is born soon after the daughter's birth. Younger siblings were treated as ‘competing' if they were a born alive (as opposed to a pre-term stillbirth) within 1.5 years of the focal female, which represents the lower quartile of interbirth intervals in this population (mean inter-birth interval for Amboseli females is 1.75 years; note that Fig. 2 gives an estimate of 2.13 years because it is calculated from the slope of the regression line between lifespan and total offspring, based only on cases in which the older offspring survives to at least age 1).

### Construction of the cumulative adversity index

The full six-factor cumulative adversity index was constructed by summing the number of adverse conditions each female experienced in early life. Each adverse condition was represented as a binarized variable, capturing whether the adverse condition occurred in the case of maternal loss, close-in-age younger sibling, and drought, and whether a female fell in the extreme quartile of the population distribution, in the case of maternal rank, maternal social connectedness and experienced density. The adversity index thus ranged from 0 to 6 (although no females in our study sample experienced more than four adverse conditions). For reduced cumulative adversity indexes, we recalculated the adversity index for the same individuals included in the full analysis of cumulative adversity, but after excluding a specified subset of factors. For example, excluding the conditions of maternal loss and close-in-age younger sibling resulted in a cumulative adversity index ranging from 0 to 4 instead of 0 to 6 (applied to all 196 females). For all analyses, we grouped females that experienced three or more adverse conditions into one category because only a small fraction of females experienced three or four adverse conditions, and none experienced five or six.

### Cox proportional hazards model for survival data

To assess the relationship between early life adversity and mortality, we fit Cox proportional hazards models, either with a single predictor variable (the cumulative adversity index, a continuous variable) or multiple predictors, with each source of early adversity modelled separately. All models were fit using the function *coxph*, and were checked for adherence to the proportional hazards assumption using the R package *survival*^60^. To assess the prediction error for the resulting survival curves, we used the function *pec* in the R package *pec*^61^, with the data split method run using bootstrap resampling (option BootCv, 100 iterations allowing sampling with replacement: Supplementary Fig. 1).

### Early adversity and social connectedness in adulthood

To investigate the relationship between a female's cumulative early life adversity and her adult social connectedness we constructed LMMs using the *lmm* function in the R package *lme4* (ref. 62). Social connectedness was measured using the same approach described above for maternal social connectedness^24^, except that we focused on the focal female's grooming patterns instead of her mother's and measured both her connectedness to other adult females and, for a separate analysis, her social connectedness to adult males (both values independently predict lifespan in the Amboseli baboon population and are themselves weakly negatively correlated^24^). Because female social connectedness varies over the life course due to changes in the availability of female kin, age and group demographics, we included multiple values for each female (one value for each year of her adult life) in the LMM, controlling for female identity as a random effect. The sample size of females for this analysis was smaller (*n*=123) than for the survival analyses because of missing data on social connectedness in adulthood (social connectedness was not estimated in group-years during which group membership was unstable, for example, during protracted fission events). In our LMMs, the response variable was the social connectedness index (either to adult females, Fig. 2a, or to adult males, Fig. 2b) calculated for each female in each year of her life where data were available. The number of adverse conditions the female experienced in early life was a fixed ordinal predictor variable (0 to ≥3 conditions, as above), and female age (in years), female ordinal dominance rank, whether the female's mother was still alive, and whether the female had adult daughters living in her social group were fit as additional fixed effects covariates (where values for the same focal female varied across the years of her life in which social connectedness measures were available).

### Data availability

Associated data sets are deposited in Dryad (doi:10.5061/dryad.5t2k7).

## Additional information

**How to cite this article:** Tung, J. *et al*. Cumulative early life adversity predicts longevity in wild baboons. *Nat. Commun.* 7:11181 doi: 10.1038/ncomms11181 (2016).

## Supplementary Material

Supplementary Information Supplementary Figures 1-2, Supplementary Tables 1-5, Supplementary Note 1 and Supplementary References.
