# Supplementary material for: Cumulative early life adversity predicts longevity in wild baboons
Source: Nat Commun. 2016 Apr 19;7:11181. doi: 10.1038/ncomms11181 (PMC4838827; doi:10.1038/ncomms11181)
Supplement: Supplementary Information — Supplementary Figures 1-2, Supplementary Tables 1-5, Supplementary Note 1 and Supplementary References. [file ncomms11181-s1.pdf]

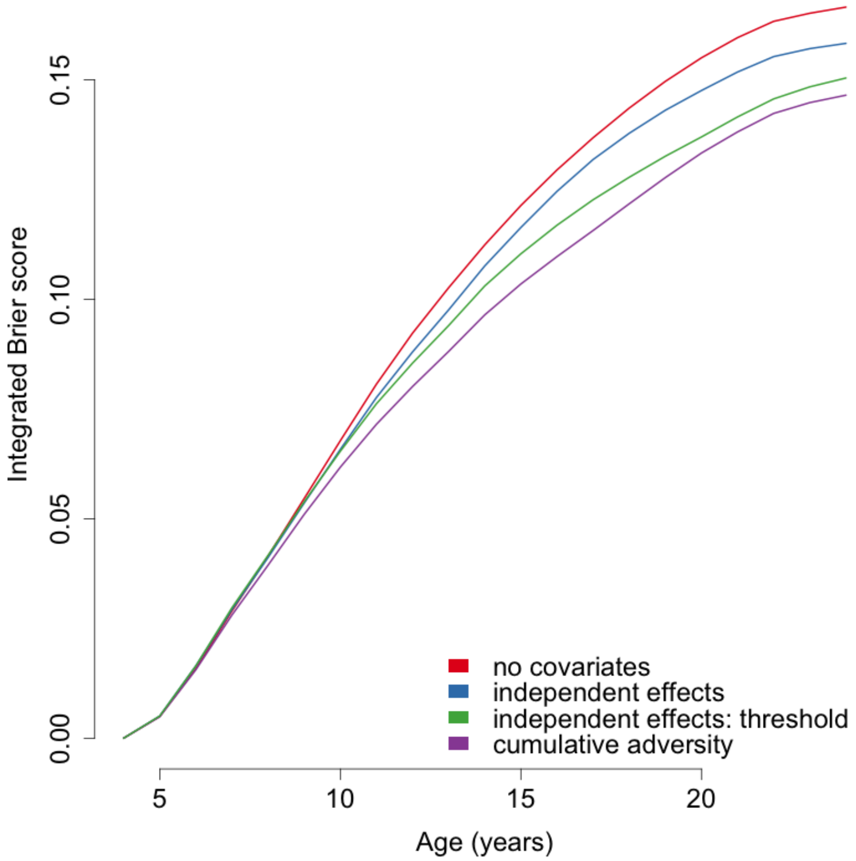

Supplementary Figure 1. Prediction error across models for early life adversity effects on survival. Integrated Brier score (IBS) measures of survival curve prediction error from age 4 to the corresponding age on the x-axis. Lower scores reflect lower prediction error (higher accuracy). To generate prediction error estimates, we conducted bootstrap resampling as follows: sample n=196 data points from the full data set with replacement to construct the training set; construct the test set from any data points not sampled; iterate this process 100 times. IBS values integrated to age 24 were 0.167 for no covariates (red line); 0.158 for the independent effects model (blue line); 0.150 for the independent effects model using binary predictors coded the same way as in the cumulative adversity model (‘independent effects: threshold:’ green line); and 0.146 for cumulative adversity (purple line). All IBS estimates were calculated using the R package *pec*[3].

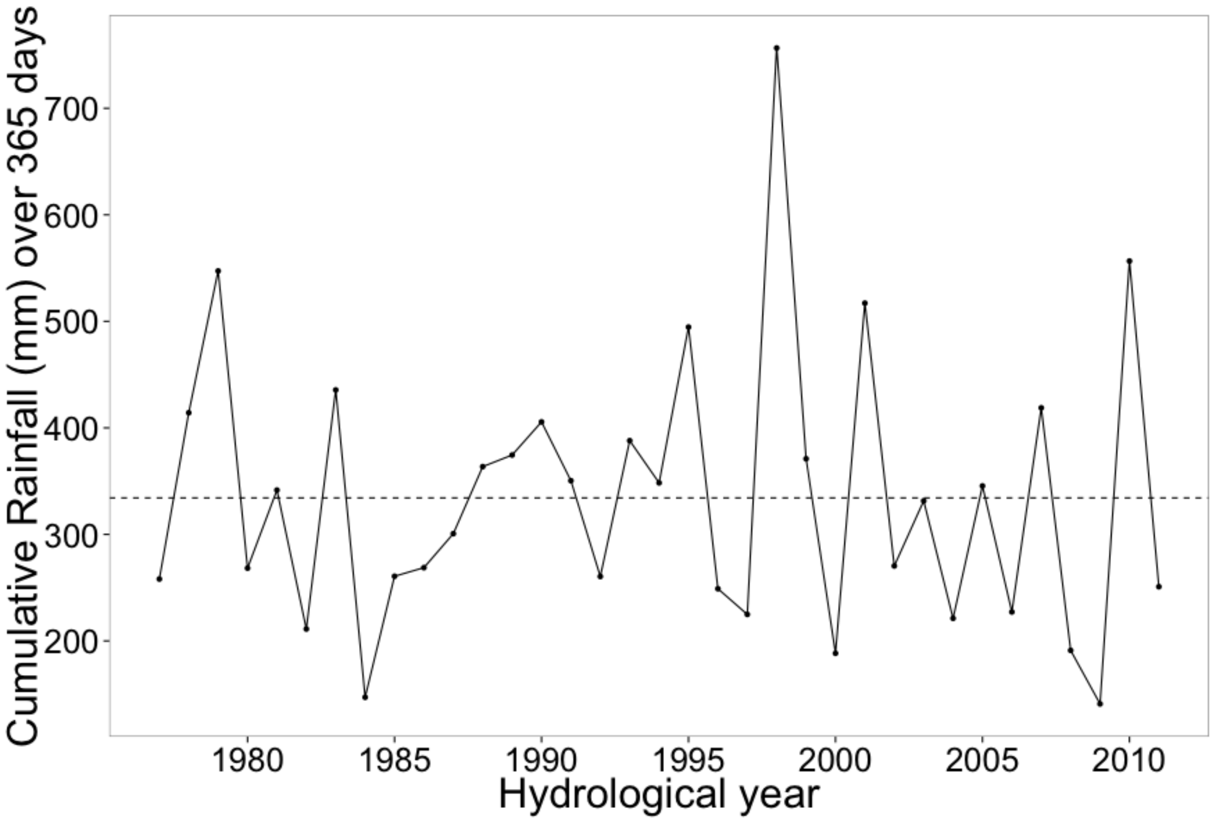

Supplementary Figure 2. Cumulative yearly rainfall varies substantially in Amboseli from year-to-year.

Supplementary Table 1. Correlations between sources of early life adversity.

r<br>(p)	maternal rank	competing younger sibling	maternal social connectedness	drought	maternal loss	experienced density
**maternal rank**	---	**-0.211**<br>**(0.003)**[1]	-0.053<br>(0.457)	0.065<br>(0.366)	-0.054<br>(0.454)	**0.438**<br>**(1.37×10^{-10})**[2]
**competing younger sibling**		---	**0.212**<br>**(0.003)**[3]	-0.089<br>(0.215)	0.061<br>(0.396)	**-0.230**<br>**(0.001)**[4]
**maternal social connectedness**			---	0.091<br>(0.204)	-0.063<br>(0.382)	-0.018<br>(0.802)
**drought**				---	0.000<br>(1.00)	**0.193**<br>**(0.007)**[5]
**maternal loss**					---	0.003<br>(0.971)
**experienced density**						---

[1]Offspring of low ranking females were slightly less likely to have competing younger siblings within 1.5 years (high ranking females tend to have shorter interbirth intervals)
[2]Only large groups contain the lowest dominance ranks (highest ordinal rank numbers), so rank values were positively correlated with group size (higher rank numbers = lower ranks). Using a proportional rank measure instead of ordinal rank breaks up this correlation (r=-0.0004) and does not change any of the qualitative results reported in the main text. However, using proportional rank approaches violations of the Cox proportional hazards assumption, so we have retained use of ordinal rank in our analyses.
[3]Females with competing younger siblings had mothers who were slightly more socially connected to other adult females. This pattern may reflect the result that females who are more socially connected have more surviving offspring[4].
[4]Females were slightly more likely to have a competing younger sibling in small groups, perhaps reflecting lower competition in small groups.
[5]First year drought was weakly correlated with large group size; this reflects a temporal pattern in which several drought years occurred during a period when groups were particularly large.

Supplementary Table 2. Independent effects model fit for multiple sources of early life adversity (whole model $r^2 = 0.11$; p = 3.802×10^{-4}, AIC = 600.74).

Variable	Coefficient	Hazard ratio (± 95% CI)	p	Interpretation
maternal rank	0.003	1.003 (0.958 - 1.050)	0.899	
**competing younger sibling**	**0.676**	**1.966** (1.069 - 3.614)	**0.030**	**Competing younger sibling predicts earlier mortality**
maternal social connectedness	-0.265	0.767 (0.509 - 1.155)	0.204	
drought	0.170	1.185 (0.551 - 2.547)	0.664	
**maternal loss**	**1.101**	**3.007** (1.793 - 5.044)	**3.03×10^{-5}**	**Maternal loss predicts earlier mortality**
experienced density	0.028	1.029 (0.992 - 1.067)	0.133	

Supplementary Table 3. Model fit for multivariate sources of early life adversity using threshold values to define adverse early life conditions (whole model r^2 = 0.14; p = 2.29 x 10^{-5}, AIC = 594.56).

Variable	Coefficient	Hazard ratio (± 95% CI)	p	Interpretation
maternal rank	0.219	1.245 (0.716 - 2.165)	0.438	
**competing younger sibling**	**0.651**	**1.920** (1.066 - 3.448)	**0.030**	**Competing younger sibling predicts earlier mortality**
**maternal social connectedness**	**0.681**	**1.976** (1.168 - 3.348)	**0.011**	**Lowest quartile of social connectedness predicts earlier mortality**
drought	0.177	1.194 (0.555 - 2.566)	0.650	
**maternal loss**	**1.041**	**2.831** (1.671 - 4.797)	**1.1 x 10^{-4}**	**Maternal loss predicts earlier mortality**
experienced density	0.522	1.686 (0.873 - 3.257)	0.120	

Supplementary Table 4. Linear mixed model predicting adult females' social connectedness to other adult females.

Variable	Coefficient (± SE)	t	p	Interpretation
**age**	**-0.027 (0.010)**	**-2.806**	**0.005**	**Older females were more socially isolated than younger females**
Mother alive vs dead	0.126 (0.071)	1.766	0.078	
**Has adult daughters**	**0.464 (0.093)**	**5.006**	**6.92×10^{-7}**	**Females with adult daughters were more socially connected than females without daughters**
Dominance rank	-0.009 (0.006)	-1.715	0.087	
**Cumulative adversity**	**-0.138 (0.048)**	**-2.861**	**0.005**	**Females that experienced more early adversity were less connected to other females during adulthood**

Supplementary Table 5. Linear mixed model predicting adult females' social connectedness to adult males.

Variable	Coefficient (± SE)	t	p	Interpretation
**age**	**0.032 (0.015)**	**2.115**	**0.035**	**Younger females were more isolated from males than older females**
Mother alive vs dead	0.151 (0.112)	1.358	0.175	
Has adult daughters	-0.159 (0.151)	-1.053	0.293	
**Dominance rank**	-0.030 (0.008)	-3.581	**4.36×10^{-4}**	**Low-ranking females were more socially isolated than high-ranking females**
Cumulative adversity	0.048 (0.071)	0.671	0.503	

Supplementary Note 1

Correlations between early adversity and adverse adult conditions

We report a strong effect of cumulative early adversity on survival in the main text. An alternative explanation for our findings is if poor quality early life environments predict poor quality adult environments, and if adult environments are in fact responsible for our findings. This explanation is unlikely for the following reasons.

First, one of the early life insults we consider is completely uncorrelated year-to-year (drought/rainfall: see Figure S2) and another has no analogue in adult baboons (competing younger sibling, where the relevant competition is for maternal investment in early life). A third major insult, maternal loss, is correlated between early life and adulthood by construction: if a female loses her mother in early life, her mother is absent throughout the remainder of her life. However, the long period of infant and juvenile dependency on mothers strongly argues that early life loss is most important. Indeed, in more than 40 years of observation, we have observed almost no cases of infant survival following maternal loss in the first year of life. In addition, in a prior analysis, the absence of a mother in adulthood did not predict adult survival[2].

Second, for the remaining three early life insults, we observe relatively weak relationships between early life conditions and mean values in adulthood. Maternal social connectedness explains only 3.9% of variance in a female's mean social connectedness (to other females) in adulthood ($p = 0.028$, $n = 123$), and group size at birth explains only 14.5% of variation in mean group size in adulthood ($p = 3.60 \times 10^{-8}$). The correlation between maternal rank and mean rank in adulthood is substantially stronger ($r^2 = 30.4\%$, $p = 1.11 \times 10^{-15}$). However, these correlations cannot account for the effects of cumulative early adversity, because group size and dominance rank (as well as maternal presence) in adulthood have no detectable effects on survival in Amboseli females ($p=0.224 – 0.618$ using a Cox proportional hazards model with time-varying covariates[2]). Indeed, the only significant effects of adult environment on survival were due to social connectedness. Our results explain one reason that adult social connectedness itself may vary: as a result of cumulative early adversity. Notably, the effects of cumulative early adversity on survival are also much larger than the effects of adult SCI alone.

Supplementary References

1. Lea, AJ, Altmann, J, Alberts, SC & Tung, J. (2015). Developmental constraints in a wild primate. *American Naturalist* **185**.
2. Archie, EA, Tung, J, Altmann, J & Alberts, SC. (2014). Social affiliation matters: both same-sex and opposite-sex relationships predict survival in wild female baboons. *Proceedings of the Royal Society* **281**, 20141261.
3. Mogensen, UB, Ishwaran, H & Gerds, TA. (2012). Evaluating random forests for survival analysis using prediction error curves. *Journal of Statistical Software* **50**, 1.
4. Silk, JB, Alberts, SC & Altmann, J. (2003). Social bonds of female baboons enhance infant survival. *Science* **302**, 1231-1234.
